# Applications of Polymer Electrolytes in Lithium-Ion Batteries: A Review

**DOI:** 10.3390/polym15193907

**Published:** 2023-09-27

**Authors:** Jayeeta Chattopadhyay, Tara Sankar Pathak, Diogo M. F. Santos

**Affiliations:** 1 Amity Institute of Applied Sciences, Amity University Jharkhand, Ranchi 834002, India; 2 Surendra Institute of Engineering and Management, Dhukuria, Siliguri 734009, West Bengal, India; drtspathak1971@gmail.com; 3 Center of Physics and Engineering of Advanced Materials, Laboratory for Physics of Materials and Emerging Technologies, Chemical Engineering Department, Instituto Superior Técnico, Universidade de Lisboa, 1049-001 Lisbon, Portugal

**Keywords:** polymer electrolytes, lithium-ion batteries, solid polymer electrolytes, gel polymer electrolytes

## Abstract

Polymer electrolytes, a type of electrolyte used in lithium-ion batteries, combine polymers and ionic salts. Their integration into lithium-ion batteries has resulted in significant advancements in battery technology, including improved safety, increased capacity, and longer cycle life. This review summarizes the mechanisms governing ion transport mechanism, fundamental characteristics, and preparation methods of different types of polymer electrolytes, including solid polymer electrolytes and gel polymer electrolytes. Furthermore, this work explores recent advancements in non-aqueous Li-based battery systems, where polymer electrolytes lead to inherent performance improvements. These battery systems encompass Li-ion polymer batteries, Li-ion solid-state batteries, Li-air batteries, Li-metal batteries, and Li-sulfur batteries. Notably, the advantages of polymer electrolytes extend beyond enhancing safety. This review also highlights the remaining challenges and provides future perspectives, aiming to propose strategies for developing novel polymer electrolytes for high-performance Li-based batteries.

## 1. Introduction

Lithium-ion batteries (LIBs) have experienced substantial growth and have become dominant in various applications, such as electric vehicles and portable devices, ever since their commercialization by Sony Corporation in 1991 [1,2,3]. Despite the advantages of LIBs, such as their high energy density and long lifespan, concerns regarding safety and their extremely high energy density have become limitations hindering further progress. The pursuit of even higher energy density has led to safety concerns, as packing more energy into a confined space can increase the risk of thermal runaway and safety incidents. Balancing energy density with safety is now a critical challenge in the battery industry. Moreover, extremely high energy density can result in more rapid capacity degradation and reduced cycle life, which affects the longevity and reliability of LIBs. In recent years, LIBs, also known as Li-ion batteries, have made rapid advancements due to their usage in grid energy storage, electric vehicles (EVs), and portable electronic devices [4]. Enhancing cycling performance and ensuring safety in LIBs heavily relies on the development of high-performance electrolyte materials. Conventional non-aqueous liquid electrolytes containing combustible organic solvents such as carbonates and ethers pose safety issues such as fire, explosion, and electrolyte leakage, presenting a challenging situation for LIB development [5]. These safety concerns become even more critical in post-LIBs, such as Li-metal batteries, lithium-sulfur (Li-S) batteries, and lithium-oxygen (Li-O_2_) batteries, which offer higher energy density [6]. Commercial LIBs currently employ liquid organic electrolytes, which possess high conductivity and excellent wetting ability on electrode surfaces. However, liquid electrolytes have inherent drawbacks, including electrochemical instabilities, potential hazards, and limited ion selectivity.

In contrast, solid electrolytes offer superior safety and thermal stability compared to liquid electrolytes since they can physically separate positive and negative electrodes, preventing thermal runaway under high temperatures or impact. Additionally, solid electrolytes effectively suppress the growth of lithium dendrites, enabling the use of a lithium metal anode. However, challenges such as inadequate ionic conductivity and insufficient interface contact must be addressed for developing solid lithium batteries [7,8,9,10]. Key requirements for solid electrolytes to meet commercial demands include excellent ionic conductivity, favorable mechanical properties, and exceptional interfacial stability with the electrodes [11].

**Scheme 1:**
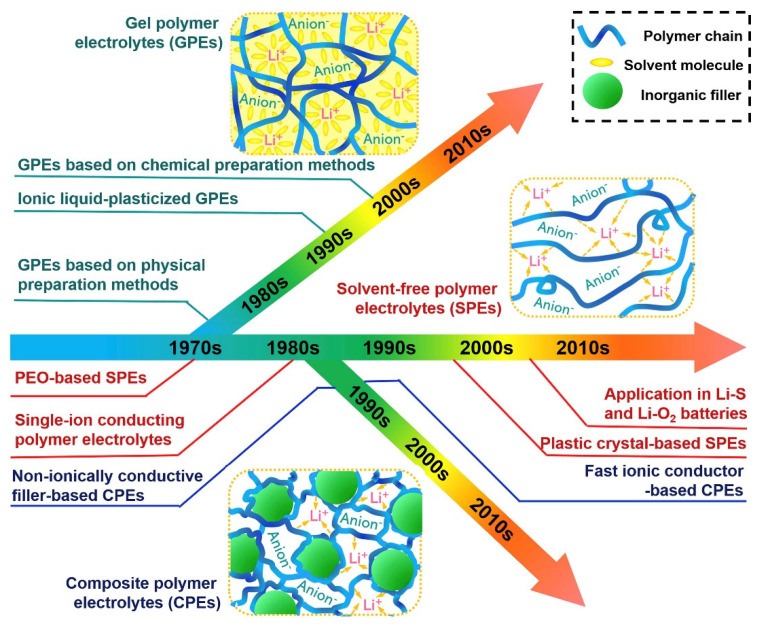
A chronological overview of the development of polymer electrolytes in lithium-ion batteries (LIBs) applications. Reprinted from [12] with permission from Elsevier.

Polymer electrolytes, a type of electrolyte used in LIBs, combine polymers and ionic salts. Their integration into LIBs has resulted in significant advancements in battery technology, including improved safety, increased capacity, and longer cycle life. The history of polymer electrolytes in LIBs dates back to the late 1990s. The first commercial application of polymer electrolytes in LIBs was in the early 2000s. Sony Corporation introduced these batteries in their consumer electronics products, marking a significant milestone in the battery industry. Sony’s introduction of the first polymer electrolyte-based LIBs represented a departure from traditional liquid electrolyte-based batteries [12]. Scheme 1 illustrates a chronological overview of the development of polymer electrolytes in the applications in LIBs.

Before introducing polymer electrolyte-based LIBs, traditional liquid electrolyte-based batteries dominated the landscape of portable energy storage solutions. These batteries primarily relied on liquid electrolytes to facilitate the flow of ions between the positive and negative electrodes, enabling the conversion of chemical energy into electrical power. Lead-acid batteries were among the earliest and most common examples of these traditional liquid electrolyte batteries. Due to their affordability and reliability, they found widespread use in applications such as automobiles and uninterruptible power supplies (UPS). However, they suffered from drawbacks such as low energy density and the need for maintenance. The era before polymer electrolyte-based LIBs was also marked by nickel-cadmium (NiCd) and nickel-metal hydride (NiMH) batteries. NiCd batteries offered good performance but were plagued by the “memory effect,” which reduced their capacity over time if not fully discharged before recharging. NiMH batteries addressed some of these issues but still had limitations in terms of energy density. The introduction of polymer electrolyte-based LIBs revolutionized the energy storage industry. These batteries combined the high energy density of lithium-ion chemistry with solid or gel-like polymer electrolytes. This innovation not only offered greater energy density and longer cycle life but also improved safety by reducing the risk of electrolyte leakage and thermal runaway. As a result, polymer electrolyte-based LIBs became the go-to choice for powering portable electronics, electric vehicles, and many other applications, reshaping how we store and use energy in our daily lives. Polymer electrolytes are now used in a wide range of applications, including consumer electronics, electric vehicles, and energy storage systems. In addition, polymer electrolytes have been used in medical and military applications, where safety and reliability are key factors. Advances in materials science and engineering have driven the development of polymer electrolytes. In particular, scientists have sought to improve the stability and ionic conductivity of the electrolytes to make them more suitable for use in LIBs. In terms of conductivity, these electrolytes have shown promising performance. For instance, certain gel-based polymer electrolytes based on polyethylene oxide (PEO) doped with lithium salts have demonstrated relatively high ionic conductivity, making them suitable for use in LIBs. This enhanced conductivity enables efficient ion transport within the electrolyte, improving battery performance and faster charge-discharge cycles. Moreover, these electrolytes exhibit good stability under various conditions. These polymer electrolytes have been designed to withstand a wide range of temperatures, ensuring their reliability in extreme environments. For example, in electric vehicles, where temperature variations can be significant, polymer electrolytes help maintain consistent battery performance. Additionally, these electrolytes are known for their chemical stability, reducing the risk of side reactions or degradation over time, which is crucial for the long-term durability of energy storage devices. Furthermore, the flexibility of these polymer electrolytes allows them to adapt to different form factors, making them ideal for use in various battery designs, from small-scale applications such as wearables to large-scale energy storage systems. This adaptability enhances the overall appeal of gel-based polymer electrolytes in the field of electrochemical energy storage [13,14]. For example, using fluorinated compounds and gel-like polymers has led to improved performance [15]. In addition, composite polymers have allowed the design of electrolytes with tailored properties.

Armand played a pioneering role in the advancement of polymer electrolytes based on polyethylene oxide-lithium salts (PEO:Li) [16,17]. In his initial publication in 1970, Armand proposed using polymer electrolytes to enhance the energy density and efficiency of LIBs. This proposal instilled confidence, leading renowned researchers worldwide to concentrate on developing polymer electrolytes as a combined separator and electrolyte for LIB applications. Polymer electrolytes possess distinctive qualities such as flexibility in size, transparency, lightweight, ease of production, elasticity, and the ability to establish efficient contact between the electrode and electrolyte.

A gel-based polymer electrolyte is a type of electrolyte that has been developed as a much safer and more efficient alternative to traditional LIBs. It is composed of a polymer matrix, usually a polymer gel, which contains a liquid electrolyte. This type of electrolyte is more stable than traditional solutions, and has a higher conductivity, allowing for faster charge and discharge times. Furthermore, it is less corrosive than traditional solutions, making it much safer. Its use in batteries has been growing, and gel and solid polymer electrolytes are now used in a range of applications, from electric vehicles to medical devices. Polymer electrolytes are an important component of LIBs. They provide an efficient ionic conduction path for the flow of lithium ions between the positive and negative electrodes. Polymer electrolytes can be classified into two main types: solid and liquid. Solid polymer electrolytes (SPEs) are composed of a polymer host material and a salt, while gel polymer electrolytes (GPEs) are an ionic liquid solution in a polymer matrix. SPEs offer good stability but low ionic conductivity. Liquid polymer electrolytes provide good ionic conductivity but are prone to liquid leakage. Additionally, both polymer electrolyte types can be further classified based on their chemical composition and preparation method.

The present review explores the recent progress in polymer electrolytes, specifically SPEs, GPEs, and composite polymer electrolytes (CPEs). The discussion covers ion-conductive mechanisms, fundamental characteristics, and synthesis strategies of these electrolyte types. Emphasis is placed on their potential applications in LIB systems. The analysis also includes examining critical studies, considering the challenges and prospects associated with polymer electrolytes in the context of their application in LIBs.

## 2. Polymer Electrolytes

**Figure 1:**
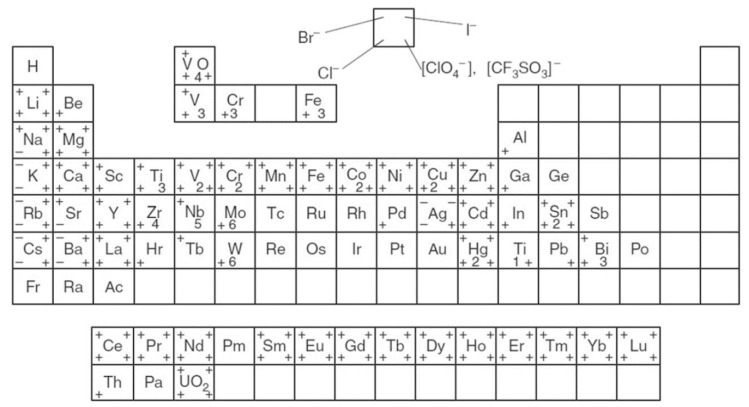
Representation of complex formation between poly(ethylene oxide) (PEO) and various metal salts. (‘+’Complex formed; ‘−’ no evidence of complex) [24]. Reprinted with permission from Springer Nature.

Polymer electrolytes emerged in the 1970s as a promising option for ion-conducting materials in energy storage and conversion devices. They offer superior stability and enhanced safety compared to organic liquid electrolytes. Liquid electrolytes, typically consisting of dissolved salts in a solvent, often exhibit higher ionic conductivities than polymer electrolytes. This superior conductivity allows for faster ion transport within the electrolyte, contributing to quicker charge and discharge rates in batteries. On the other hand, polymer electrolytes, which consist of solid or gel-like polymers infused with ionic salts, tend to have lower ionic conductivities. However, advancements in polymer electrolyte technology have led to the development of highly conductive materials, particularly important for solid-state batteries. Polymer electrolytes combine the safety advantages of solid electrolytes with improved ionic conductivity, offering a promising alternative to traditional liquid electrolytes in terms of both performance and safety. These polymer materials are gaining prominence in the scientific community as an emerging class of solid-state ionic conductors (SSICs). While fast ionic conductors are typically made of inorganic materials with excellent transport properties, their use as both electrolytes and electrode separators is limited due to low electrolyte stability. Inorganic solid electrolytes, while promising for certain energy storage and electronics applications, can be inherently unstable for several reasons. Firstly, many inorganic materials used as solid electrolytes are sensitive to moisture and air, leading to degradation over time. Exposure to humidity can result in the formation of unwanted chemical compounds or phase changes, compromising the electrolyte’s integrity and ionic conductivity. Secondly, inorganic solid electrolytes may be susceptible to mechanical stress and strain, especially in high-energy-density applications such as solid-state batteries. Cracks or defects can form in the solid electrolyte, reducing its stability and long-term performance. A flexible and soft material is required as a separator to accommodate volume variations during charging and discharging. In polymer electrolytes, ionic conduction occurs at the glass transition temperature (T_g_), and the relatively low conductivity is compensated for by the electrolyte’s form, size, and flexibility. Multivalent cations were preferred due to their easier handling and lower cost than alkali metal salts. However, the presence of crystalline and amorphous phases in these electrolytes complicated the accurate study of their characteristics [18,19]. In 1973, Peter V. Wright and D.E. Fenton introduced research on ionic conductivity in polymers using PEO with alkali iodide ions [20]. Subsequently, in the same year, Armand utilized a combination of PEO and alkaline salt as an ionic conductor in batteries. Various electrolytes were synthesized based on the lattice/dissociation energy of the dopant salt, utilizing salt and polymer hosts. In systems with liquid electrolytes, the electrolyte, together with a separator, serves as a medium for ion transport and electrode isolation. In solid-state LIBs, the polymer electrolyte functions as a thin-film membrane for both ion conduction and electrode separation. The preparation of polymer electrolytes is based on the anion’s delocalized charge, which is directly related to the lattice energy of the associated salt [21]. Polymer electrolytes are created by dissolving Li-based salt in a high molecular weight polymer host, such as PEO, resulting in dual ionic conductors. However, the combined movement of cations and anions may lead to concentration polarization, which can degrade battery performance [22,23]. To address this issue, a single ion conductor, either a cation or an anion, can be employed as the charge carrier. Most studies focus on Li cations due to their smaller size. Figure 1 illustrates the wide range of salts from the periodic table that can be doped into a polymer host to generate SPEs. The polymer chain acts as a solvent for the salt, causing its dissociation and enabling the behavior of the electrolyte [24].

Polymer electrolytes comprise a large molecule framework containing a dissolved salt with low lattice energy. This salt is dissolved in organic solvents with a low viscosity and high dielectric constant. These solvents possess desirable properties such as good ionic conductivity, high chemical stability, affordability, and safety. The underlying principle of ionic conduction in polymer electrolytes involves the formation of covalent bonds between the polymer backbones and ionizing groups. Initially, the polymer’s electron donor component interacts with the cationic part of the dopant salt, aiding in ion separation [25,26]. This process leads to an ionic hopping mechanism, resulting in the generation of ionic conductivity. For the successful formation of a complex between the polymer and salt, it is important that the salt’s lattice energy is small and that the host polymer has a high dielectric constant (κ). Ionic conduction in polymer electrolytes occurs due to the rapid motion of the polymer matrix combined with strong Lewis-type acid–base interactions between the cation and the donor atom. However, safety issues often arise when a Li metal anode is used in conjunction with liquid electrolytes. These issues are associated with the formation of irregular metal deposits during the recharging process, which can lead to the development of Li dendrites. These dendrites can cause internal short circuits due to their thread-like structure between the electrodes, potentially resulting in uncontrolled thermal runaway and rapid discharge. Using lithium ion-conducting polymer electrolytes could address some of the safety concerns when using liquid electrolytes. Polymer electrolytes offer reduced flammability and increased resistance to dendrite formation.

Nevertheless, their use is hindered by lower conductivities compared to those of liquid electrolytes at room temperature [26,27]. Polymer electrolytes possess numerous advantages, such as exceptional ionic conductivity, high energy density, absence of solvents, structural stability, minimal volatility, a wide range of electrochemical stability, ease of formation, and lightweight composition. Polymer electrolytes comprise salts dissociating within a polymer host, including electron donor groups. These electrolytes serve a dual purpose as separators and electrolytes in solid-state configurations. This dual functionality is crucial for the efficient and safe operation of these advanced battery designs. As a separator, the polymer electrolyte physically separates the positive and negative electrodes, preventing direct contact and potential short-circuits. Simultaneously, as an electrolyte, it facilitates the movement of ions, such as lithium ions in the case of LIBs, between these electrodes during the charge and discharge cycles. For polymer electrolytes to fulfil these dual roles effectively, they must possess a range of essential electrochemical characteristics. These characteristics include high ionic conductivity to enable efficient ion transport, ensuring the battery can charge and discharge quickly.

Moreover, they should exhibit good mechanical properties to maintain physical integrity and prevent dendrite growth, which can lead to safety hazards. Additionally, stability over a wide range of temperatures and the ability to withstand mechanical stress are critical features. Balancing these characteristics is essential to achieving the overall performance and safety requirements of solid-state batteries, making the development and optimization of polymer electrolytes a vital area of research in advanced energy storage technologies.

Polymer electrolytes offer a solution to many of the limitations that liquid electrolytes face, including their inflexibility in shape, size, and weight. The amorphous phase of polymer electrolytes enables fast ion transport, exhibiting conductivity levels two to three orders of magnitude higher than the crystalline phase. In recent years, SPEs have gained significant importance due to their wide-ranging applications in rechargeable lithium-ion polymer batteries, fuel cells, and electrochromic display devices, as well as in industries such as electric vehicles, aerospace, automobiles, and electronics [28]. Although various host polymers such as poly(acrylonitrile), poly(propylene oxide), poly(methyl methacrylate), and poly(maleic anhydride styrene) have been studied for the preparation of polymeric electrolytes, high molecular weight PEO-based polymer electrolytes are considered the most promising candidates for the polymer matrix. This is due to their excellent solvation capacity and ion transport mechanism [29,30]. PEO, when synthesized with high molecular weight, possesses a unique ability to dissolve lithium salts and create a homogeneous, conductive medium within the polymer matrix. This high solvation capacity ensures efficient ion transport, facilitating the movement of lithium ions between the anode and cathode during charge and discharge cycles.

Furthermore, PEO-based polymer electrolytes exhibit an advantageous ion transport mechanism. The polymer’s molecular structure allows for the formation of coordination complexes with lithium ions, providing a pathway for rapid ion conduction. This coordination mechanism enables enhanced ionic conductivity, a critical factor in the overall performance of batteries. The excellent solvation capacity and the inherent ion transport mechanism make high molecular weight PEO-based polymer electrolytes highly promising materials for energy storage applications. They offer the potential for improved battery performance, including higher energy density, longer cycle life, and enhanced safety due to their non-flammable nature. As research in this field continues to advance, PEO-based polymer electrolytes are likely to play a pivotal role in developing next-generation battery technologies, addressing the increasing demand for more efficient and reliable energy storage solutions. Extensive literature research categorizes polymer electrolytes into three main types, each described below.

### 2.1. Solid Polymer Electrolytes (SPEs)

SPEs are typically composed solely of polymer matrices and Li salts as solutes, without including liquid solvents as plasticizers. They can be easily created using solvent casting, hot molding, or extrusion techniques [31,32,33]. For a polymer matrix to be suitable for use in SPEs, it must meet several essential criteria. These criteria include:

1. Nature of the cation solvation: The interactions between the polymer and the cations should strike a balance between being strong enough to ensure salt solubility through cation solvation, while also being labile enough to promote ionic hopping between coordinating sites.
2. Dielectric constant value: The polymer host should possess a high dielectric constant, facilitating effective charge separation of the salts. This, in turn, leads to a high concentration of charge carriers.
3. Flexibility of backbone structure: The polymer chains should exhibit high flexibility in reducing the energy barrier for bond rotation. This flexibility enables the segmental motion of the polymer chains.
4. Higher molecular weight value: A high molecular weight of the polymer matrix is also desirable as it enhances the mechanical strength of the resulting SPEs.
5. The significant physical properties of various types of SPEs contribute to their suitability in advanced energy storage systems. These materials typically exhibit excellent mechanical flexibility, allowing their integration into diverse form factors, from thin-film batteries to three-dimensional structures. SPEs often possess high thermal stability, ensuring their safe operation over a wide range of temperatures. Their ability to maintain structural integrity in extreme conditions enhances the overall safety of energy storage devices. Furthermore, these electrolytes are renowned for their non-flammable nature, a stark contrast to some traditional liquid electrolytes, which mitigates the risk of fire or explosion in battery applications. Their robust chemical stability and resistance to leakage further reinforce their appeal for use in next-generation batteries, particularly in solid-state and flexible electronics. Table 1 represents the significant properties of various types of SPEs.

#### 2.1.1. PEO-Based SPEs

**Figure 2:**
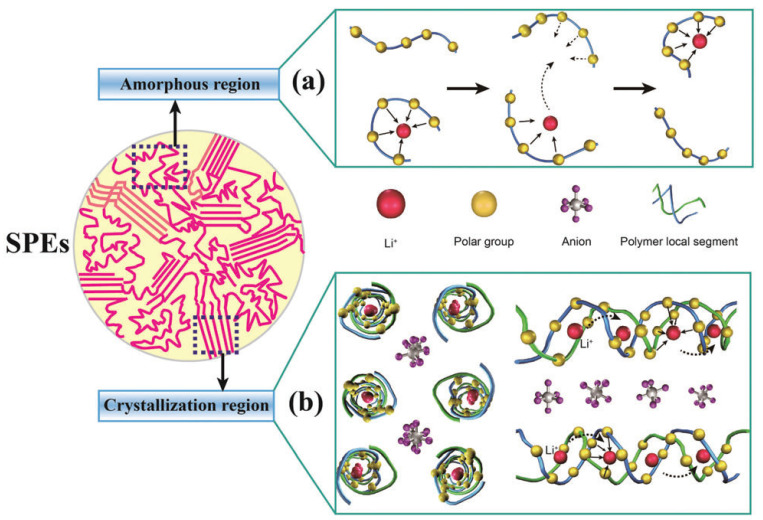
Schematic diagram on conduction mechanism in two regions of solid polymer electrolytes (SPEs) [59]. Reproduced with permission from the Royal Society of Chemistry.

Polyethylene oxide (PEO) is the most commonly used polymer matrix among all the SPEs. In SPEs based on PEO, the transportation of Li ions within the polymer matrix follows a widely accepted mechanism. This mechanism is illustrated in Figure 2, where ions dissociate from the counterions and coordinate with electron-donor groups in the polymer host [56]. Generally, an ideal SPE system incorporates a salt characterized by a low lattice energy and a host polymer with a high dielectric constant. This combination is chosen to enhance the efficient dissociation and transport of ions within the electrolyte [57]. The crystalline polymer chains naturally arrange themselves into interlocking cylinders or channels within the structured framework. Lithium ions find their place within these channels, while the anions are situated externally and remain uncoordinated with the lithium ions [58]. This is supported by the X-ray structure of the P(EO)_6_–LiAsF_6_ complex, which reveals that Li ions are solvated by PEO sheaths. Under the influence of an electric field, these cations tend to hop from one coordinating site (usually consisting of more than three electron donor groups) to another. This ion hopping is facilitated either by the segmental motion of the polymer chains or by an ion-cluster-assisting function, where temporary re-association with the counterion occurs before being resolvated by electron donor groups in the polymer. It is generally believed that ionic conduction in PEO-based SPEs primarily takes place in the amorphous region of the PEO matrix, while the crystalline part exhibits limited ion motion. Therefore, the ionic conductivity of PEO-based SPEs heavily relies on the polymer matrix’s ability to solvate Li salts and the ratio of crystalline phase to amorphous phase. Since the PEO chain is mostly crystalline below 65 °C, PEO-based SPEs typically exhibit low ionic conductivities in the range of 10^−8^ to 10^−7^ S cm^−1^ at room temperature. This limited ionic conductivity significantly restricts the practical application of PEO-based SPEs.

Consequently, various modification strategies are widely employed to enhance the ionic conductivity of PEO-based SPEs. These strategies include structural modifications of the polymer, optimization of the Li salt structure and proportion, and introduction of organic plasticizers or inorganic fillers, among others. This discussion focuses on advancements in PEO-based single-ion conducting polymer electrolytes (SIPEs), while other modification strategies will not be extensively covered, as they have been discussed in recent reviews. Wetjen et al. reported quaternary polymer electrolyte membranes comprising PEO, lithium bis(trifluoromethanesulfonyl)imide (LiTFSI), N-alkyl-N-butylpyrrolidinium bis(trifluoromethanesulfonyl)imide (Pyr_A,4_TFSI) as an ionic liquid, and a SiO_2_ filler. Differential scanning calorimetry results showed that adding SiO_2_ and different ionic liquids decreased the PEO melting enthalpy, increasing the ionic conductivity and the Li transference number [59]. In another work, they introduced uniformly distributed ZnO nanoparticles within a smooth electrolyte film, which increased the thermal and electrochemical stability during the application in solid-state lithium-metal batteries [60].

#### 2.1.2. Plastic-Based SPEs

Plastic crystals are a type of material that exhibits order in the arrangement of their positions but disorder in their orientations within a specific temperature range due to the rotational motions of molecules or ions. These materials include inorganic salts (such as Li_2_SO_4_ and a-Na_3_PO_4_), molecular species (such as succinonitrile (SN) and sebaconitrile), and organic ionic plastic crystals (OIPCs). Plastic crystals have higher diffusivity and plasticity than traditional rigid crystals, making them suitable for developing solid electrolytes composed of Li salts dissolved in plastic crystals. Consequently, these electrolytes demonstrate significantly enhanced conductivity. SN is an example of a molecular plastic crystal that exhibits plastic crystal behavior between approximately −35 °C and −62 °C. The high polarity of SN molecules allows for a high solubility of Li salts, making them ideal for use as solid electrolytes in LIBs [61,62]. In 2004, Alarco et al. reported that the ionic conductivity of 5 mol% LiTFSI in SN as a solid electrolyte exceeded 3 × 10^−3^ S cm^−1^ at 25 °C, two orders of magnitude higher than that of traditional SPEs. These SN-based solid electrolytes also demonstrated favorable thermodynamic stability (up to 160 °C), high resistance against electrochemical oxidation (up to 6 V vs. Li^+^/Li), and high charge-discharge efficiencies in Li_4_Ti_5_O_12_//LiFePO_4_ or LiCoO_2_ cells. However, the mechanical strength of these solid electrolytes is limited (maximal tensile stress of 0.2 MPa), hindering their practical application. To address this issue, high-strength polymers are incorporated into SN-based solid electrolytes to develop SPEs with high ionic conductivity and sufficient mechanical strength [63,64].

**Figure 3:**
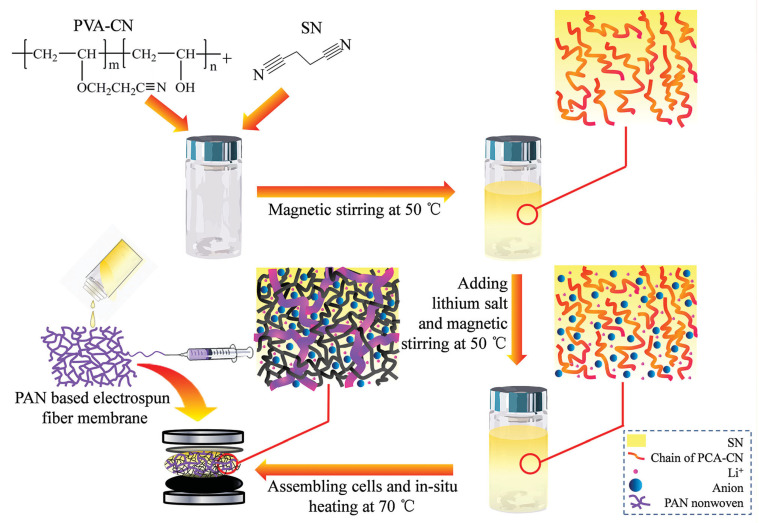
Schematic diagram for the in-situ synthesis process of solvent-free electrolyte based on nitrile materials [65]. Reproduced with permission from Wiley-VCH.

Zhou et al. introduced a hierarchical solvent-free electrolyte based on nitrile materials (SEN), which was prepared by in situ polymerization of a precursor solution containing cyanoethyl polyvinyl alcohol (PVA-CN) in an SN-based solid electrolyte within a polyacrylonitrile (PAN)-based electrospun fiber membrane (Figure 3). The cross-linked PVA-CN framework significantly enhanced the mechanical strength of the SN-based solid electrolyte, even above its melting point, maintaining a quasi-solid state. The PAN-based electrospun fiber membrane allowed for a thinner electrolyte film, resulting in improved strength. The developed SEN film exhibited high ionic conductivity (3.02 × 10^−3^ S cm^−1^), high mechanical strength (15.31 MPa), and good flexibility, allowing it to be repeatedly bent at room temperature. However, the poor compatibility between nitriles and Li metal, leading to side reactions such as self-polymerization, still hampers the practical application of SN-based SPEs in Li-metal batteries [65].

#### 2.1.3. Polysiloxane-Based SPEs

Polysiloxane-based SPEs are a class of materials that have gained significant attention in the field of electrochemistry and energy storage. These SPEs exhibit unique properties and have shown great potential for LIBs, fuel cells, and supercapacitor applications. Polysiloxanes are organic–inorganic hybrid polymers composed of repeating siloxane (-Si-O-) units, providing excellent thermal and chemical stability. These materials can be tailored to possess high ionic conductivity by incorporating appropriate ionic species, such as lithium or proton carriers. The presence of these ionic species facilitates the transport of ions within the solid matrix, enabling efficient charge transfer. One of the key advantages of polysiloxane-based SPEs is their flexibility and mechanical robustness. They can be designed to have a wide range of mechanical properties, allowing for their incorporation into various device architectures [66]. Moreover, their high thermal stability ensures the integrity of the electrolyte even under elevated temperatures. Another noteworthy feature of polysiloxane-based SPEs is their excellent electrochemical stability, contributing to prolonged cycle life and enhanced safety in energy storage devices. They effectively suppress the formation of lithium dendrites in LIBs, preventing short circuits and improving the overall performance and lifespan of the battery. Furthermore, these SPEs offer good compatibility with electrode materials, facilitating efficient charge transfer at the electrode–electrolyte interface. This characteristic is crucial for improving the overall electrochemical performance of energy storage systems.

In conclusion, polysiloxane-based SPEs are promising materials for advanced energy storage applications. Their unique combination of mechanical, thermal, and electrochemical properties makes them an attractive choice for developing next-generation batteries, fuel cells, and supercapacitors with improved performance, safety, and durability. They play a crucial role in enhancing the performance and safety of LIBs. These SPEs act as ion-conductive membranes, facilitating the transport of lithium ions between the cathode and anode. The high ionic conductivity of polysiloxane-based SPEs enables faster ion diffusion, resulting in improved battery efficiency and higher power density.

**Figure 4:**
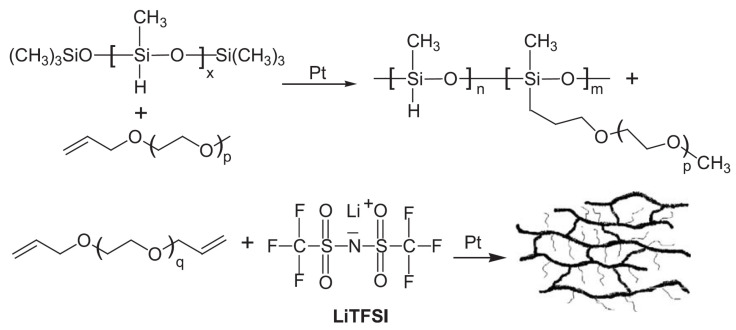
Synthesis of cross-linkable precursors and cross-linked polysiloxane network polymer electrolytes [67]. Reproduced with permission from Elsevier.

Moreover, their mechanical flexibility helps suppress the formation of lithium dendrites, which can cause short circuits and battery failure. These SPEs also exhibit excellent thermal stability, reducing the risk of thermal runaway and enhancing the safety of LIBs. Their compatibility with electrode materials allows for improved charge transfer, enhancing cycling stability, and longer battery lifespan. Zhang et al. introduced a poly(siloxane-gethylene oxide)-based interpenetrating network-type SPE for LIBs. In this diagram (Figure 4), the network polymer electrolyte demonstrates a room temperature ionic conductivity of up to 1.62 × 10^−4^ S cm^−1^. The conductivity depends on factors such as the number of repeating units of internal oligoethylene oxide, the chain length of the cross-linker, and the cross-linking density [67]. Liang et al. reported the synthesis of polysiloxane-based single-ion conductor (PSiO) via a simple thiol-ene reaction, yielding flexible and self-standing polymer electrolyte membranes (PSiOM) when blended with poly(vinylidene fluoride-co-hexafluoropropylene) (PVdF-HFP) [68].

#### 2.1.4. Polycarbonate-Based SPEs

Polycarbonate-based SPEs have emerged as promising materials for enhancing the performance and safety of LIBs. Polycarbonate is a type of thermoplastic polymer known for its high mechanical strength, excellent thermal stability, and good processability, making it suitable for various applications, including battery technology.

One of the key roles of polycarbonate-based SPEs in LIBs is to act as a medium for ion transport between the cathode and anode. These SPEs incorporate lithium salts to enable the conduction of lithium ions, facilitating efficient charge transfer during battery operation. The high ionic conductivity of polycarbonate-based SPEs ensures fast ion diffusion, leading to improved battery performance, including enhanced power density and cycling stability. They also offer excellent mechanical properties, such as toughness and flexibility. These characteristics help prevent the formation of lithium dendrites, which can cause short circuits and compromise battery safety. The flexibility of these SPEs allows them to accommodate the volume changes that occur during charging and discharging cycles, reducing mechanical stress on the electrode materials and contributing to prolonged battery lifespan [69,70].

Additionally, the high thermal stability of polycarbonate-based SPEs enhances the safety of LIBs. These SPEs exhibit resistance to thermal decomposition and have high glass transition temperatures, reducing the risk of thermal runaway and improving the overall thermal management of the battery system. The processability of polycarbonate-based SPEs enables their easy integration into battery manufacturing processes. They can be cast into thin films or directly coated onto electrode surfaces, forming a solid and uniform electrolyte layer. This simplifies the battery assembly and improves the overall battery performance. This kind of SPEs plays a crucial role in LIBs by providing efficient ion transport, mechanical protection against dendrite formation, thermal stability, and processability. These SPEs offer a promising solution for developing high-performance and safe energy storage systems, enabling advancements in various applications, including electric vehicles, portable electronics, and renewable energy storage.

**Figure 5:**
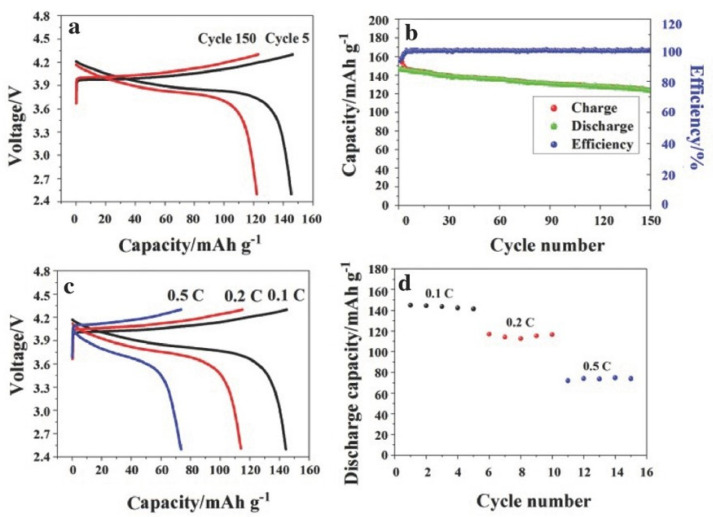
(**a**) Charge/discharge profiles of LiCoO_2_/Li cells employing PVCA-SPE after 5 and 150 cycles at 50 °C within a voltage window of 2.5-4.3 V. (**b**) Specific discharge capacity of LiCoO_2_/Li cells utilizing PVCA-SPE at constant current density of 0.1 C across multiple cycles within a voltage range of 2.5-4.3 V. (**c**) Charge and discharge characteristics of LiCoO_2_/Li cells with PVCA-SPE at varying current densities at 50 °C. (**d**) Discharge capacity of LiCoO_2_/Li cells employing PVCA-SPE under different current density conditions. Reprinted from [71] with permission from John Wiley & Sons.

Chai et al. conducted a study where they presented a SPE based on polyvinyl carbonate (PVCA) synthesized through the in situ polymerization of vinylene carbonate (VC) monomer (Figure 5) [71]. The PVCA-based SPE demonstrated an impressive ionic conductivity of 9.82 × 10^−5^ S cm^−1^ at a temperature of 50 °C. It also exhibited an electrochemical stability window of up to 4.5 V vs. Li^+^/Li, enabling excellent cycling performance in high-voltage Li/PVCA-based SPE/Li_x_CoO_2_ batteries. In simpler terms, the researchers developed an SPE by polymerizing vinylene carbonate monomer to create polyvinyl carbonate. This SPE showed a high conductivity of ions, allowing for efficient charge transfer. It also exhibited a wide voltage range that remained stable during electrochemical reactions, crucial for maintaining battery performance. This stability enabled the PVCA-based SPE to successfully integrate into high-voltage lithium batteries with a cobalt oxide cathode, resulting in excellent cycling performance. The findings from this study highlight the potential of PVCA-based SPEs as a promising electrolyte material for high-voltage LIBs. The high ionic conductivity and electrochemical stability of PVCA-based SPEs contribute to enhanced battery performance, including improved cycling efficiency and capacity retention. These results contribute to developing safer and more efficient energy storage systems for various applications, from portable electronics to electric vehicles. Allyl ether-functional polycarbonates have been synthesized by Brandell et al. using organocatalytic ring-opening polymerization of a cyclic carbonate monomer, namely 2-allyloxymethyl-2-ethyltrimethylene carbonate [72]. These polycarbonates were then utilized to create non-polyether polymer electrolytes. Mechanically stable electrolytes were obtained by subjecting the allyl side groups to UV-crosslinking, offering improved molecular flexibility with a T_g_ below −20 °C. The presence of the allyl ether side groups contributed to higher ionic conductivity in the electrolytes, reaching up to 4.3 × 10^−7^ S cm^−1^ at 25 °C and 5.2 × 10^−6^ S cm^−1^ at 60 °C. This increase in ionic conductivity can be attributed to the plasticizing properties of the allyl ether groups, which facilitate the movement of ions within the electrolyte. The effectiveness of these electrolytes was further demonstrated in thin-film lithium battery cells, indicating their potential applicability as conductive materials in practical battery devices. The combination of improved mechanical stability, enhanced molecular flexibility, and higher ionic conductivity makes these allyl ether-functional polycarbonates promising candidates for use in advanced polymer electrolytes for various electrochemical applications. In another work by the same research group, the ionic transport behavior of SPEs based on poly(trimethylene carbonate) (PTMC) and its copolymer with ε-caprolactone (CL) was investigated using a combination of experimental and computational methods [73]. FTIR spectra analysis revealed that in P(TMC20CL80) copolymer SPE, there is preferential local coordination between Li^+^ ions and ester carbonyl oxygen atoms. This suggested that specific interactions between the lithium ions and the ester groups in the copolymer play a role in the ion transport behavior. Diffusion nuclear magnetic resonance (NMR) studies showed that the copolymer SPE exhibits higher ion mobilities than PTMC, indicating that incorporating ε-caprolactone enhances the polymer electrolyte’s ionic transport properties. Both PTMC and the copolymer systems exhibit locally oriented polymer domains, which are a few hundred nanometers in size and have limited connections between them. This finding was inferred from NMR spin relaxation and diffusion data, suggesting the presence of distinct regions with differing ion transport characteristics. Potentiostatic polarization experiments demonstrated notably higher cationic transference numbers in the polycarbonate-based SPEs when compared to conventional polyether-based SPEs. This indicates that polycarbonate-based electrolytes exhibit a higher fraction of mobile cations, which is advantageous for their performance in practical applications. Furthermore, molecular dynamics simulations provided atomic-scale insights into the structure and dynamics properties of the SPEs. The simulations confirmed the preferential coordination between Li^+^ ions and carbonyl oxygen atoms, with a particular affinity for the ester-based monomers. Additionally, simulation and experiment results suggest a coupling between the dynamics of Li^+^ ions and the motion of the polymer chains, indicating that the dynamics of the polymer matrix influence the mobility of lithium ions. Overall, this comprehensive study sheds light on the ionic transport mechanisms in PTMC and its copolymer with CL-based SPEs, providing valuable information for designing and optimizing advanced SPEs for various electrochemical devices.

### 2.2. Gel Polymer Electrolytes (GPEs)

Gel-based polymer electrolytes have gained significant attention in energy storage due to their unique properties and potential applications in various electrochemical devices. These electrolytes consist of a polymer matrix that is swollen with a liquid electrolyte, resulting in a gel-like structure. The gel serves as a medium for ion transport, enabling efficient charge transfer in batteries, fuel cells, and supercapacitors. There are two main types of gel-based polymer electrolytes: physical gels and chemical gels. Physical gels are formed through the physical entanglement or cross-linking of polymer chains without chemical reactions, e.g., LiClO_4_/EC/PC in poly(methyl methacrylate) (PMMA) [74,75,76]. These gels exhibit reversible gelation and undergo sol–gel transitions with temperature or mechanical stress. Physical gels offer easy preparation, flexibility, and good mechanical stability. On the other hand, chemical gels are formed through chemical reactions, such as polymerization or cross-linking reactions. Chemical gels have a permanent gel structure and are generally more mechanically robust than physical gels. These gels offer improved stability and can be tailored to have specific properties by adjusting the chemical composition and cross-linking density. Both physical and chemical gels provide several benefits for polymer electrolytes. They offer high ionic conductivity due to the presence of a liquid electrolyte within the gel matrix. The gel structure immobilizes the liquid electrolyte, preventing its leakage and improving the safety of electrochemical devices. Moreover, the gel matrix can accommodate volume changes during charge-discharge cycles, reducing mechanical stress on the electrodes and enhancing the device’s lifespan. Therefore, gel-based polymer electrolytes, whether physical or chemical gels, are versatile materials with significant potential for energy storage applications. Their unique combination of ionic conductivity, mechanical stability, and safety features makes them promising candidates for developing advanced electrochemical devices with improved performance and durability. Gel-based polymer electrolytes have emerged as promising materials for enhancing the performance and safety of LIBs. These electrolytes, consisting of a polymer matrix swollen with a liquid electrolyte, play a crucial role in facilitating the transport of lithium ions between the cathode and anode. One of the key roles of gel-based polymer electrolytes in LIBs is to provide a stable and conductive medium for ion transport. The gel structure immobilizes the liquid electrolyte, preventing leakage and enabling efficient charge transfer. This improves battery performance, including enhanced ionic conductivity, higher power density, and better cycling stability. Moreover, the gel matrix of these electrolytes can accommodate the volume changes that occur during the lithium-ion insertion and extraction processes. This flexibility helps mitigate mechanical stress on the electrode materials, reducing the risk of electrode degradation, and enhancing the battery’s overall lifespan.

Gel-based polymer electrolytes also contribute to the safety of LIBs. The gel matrix helps prevent the formation of lithium dendrites, which can cause short circuits and compromise battery integrity. Additionally, the immobilized liquid electrolyte reduces the flammability and volatility associated with traditional liquid electrolytes, minimizing the risk of thermal runaway and enhancing the overall safety of the battery system. Furthermore, the processability and flexibility of gel-based polymer electrolytes allow for their easy integration into battery manufacturing processes. They can be cast into various shapes and sizes, enabling their use in different battery designs and configurations. In summary, gel-based polymer electrolytes play a crucial role in LIBs by providing a stable and conductive medium for ion transport, accommodating volume changes, enhancing safety, and allowing for versatile battery designs. These electrolytes offer a promising solution for developing high-performance and safe energy storage systems, contributing to advancements in portable electronics, electric vehicles, and renewable energy applications.

Santiago et al. recently reported a GPE made with polyethylene glycol dimethyl ether (PEGDME) as the plasticizer had a significantly lower flammability than a conventional GPE made with ethylene carbonate (EC) as the plasticizer. The PEGDME-based GPE had a flash point of 120 °C, while the EC-based GPE had a flash point of 60 °C. This means the PEGDME-based GPE is less likely to catch fire than the EC-based GPE [77]. Jie et al. reported the excellent electrochemical stability and high dielectric constant of poly(vinylidenefluoride-co-hexafluoropropylene) (PVDF-HFP) based GPEs. A safe solvent (N-methyl-2-pyrrolidone) with higher boiling (203 °C) and flash points (95 °C) was utilized to fabricate a flexible PVDF-HFP-based GPE [78]. In general, GPEs are less flammable than liquid electrolytes. This is because the polymer matrix in the gel electrolyte helps disperse the liquid solvent, making it less likely to vaporize and catch fire.

However, GPEs are still flammable, and their flammability can be affected by the choice of polymer matrix, solvent, and additives. SPEs are generally considered to be safer than GPEs. This is because SPEs do not contain any liquid solvents, the main source of flammability in GPEs. However, SPEs also have lower ionic conductivities than GPEs, which can limit their performance in batteries. The flammability and safety of GPEs have been measured and presented in the literature in various ways. One common method is to measure the flash point of the electrolyte. The flash point is the temperature at which the electrolyte will ignite in the presence of an open flame. Another standard method is to measure the oxygen index of the electrolyte. The oxygen index is the percentage of oxygen in the air required to support the electrolyte’s combustion. A higher oxygen index indicates a more flame-resistant electrolyte. In addition to these methods, the flammability and safety of GPEs can also be assessed by measuring their thermal stability, thermal decomposition products, and ability to suppress lithium dendrite formation.

Polymers in GPEs can also be tailored to improve compatibility with electrode materials. By optimizing the polymer composition and structure, the interface between the polymer electrolyte and electrodes can be enhanced, leading to better charge transfer and electrochemical performance. Mecerreyes’ group reported that single-ion polymer electrolytes have played an important role in developing the next-generation lithium metal batteries [79]. They performed the synthesis and characterization of single-ion conducting polyurethanes (SIPUs) based on PEG and a specifically designed ionic liquid monomer (bis-MPTFSI) [80]. The activities of GPEs based on SIPUs for lithium-metal batteries operating at room temperature have been investigated (80 cycles at C/10 with nearly 100% efficiency), which have been depicted as one of the first examples of polyurethane-based poly(ionic liquid)s for application in battery science [80]. Forsyth et al. developed a novel GPE based on a combination of a polymeric ionic liquid (polyIL) called poly(diallyldimethylammonium) bis(trifluoromethanesulfonyl)imide (PDADMA TFSI), and a high concentration phosphonium ionic liquid known as trimethyl(isobutyl)phosphonium bis(fluorosulfonyl)imide (P_111i4_FSI) [81]. These researchers investigated the behavior of these GPEs by utilizing various analytical techniques such as differential scanning calorimetry, electrochemical impedance spectroscopy, and solid-state NMR. Additionally, they studied the impact of introducing Al_2_O_3_ nanoparticles on the properties of the polymer electrolyte. The results showed that incorporating high lithium-concentration ionic liquids into the polyIL effectively lowered the T_g_ of the resulting GPE. This, in turn, leads to improved ion dynamics and higher ionic conductivity, making the electrolyte more conducive for efficient ion transport. The results showed a high ionic conductivity of 0.28 mS cm^−1^ at 30 °C when the GPEs contained 5 wt.% Al_2_O_3_ and 50 wt.% of ionic liquid. The conductivity increased with an increased ionic liquid content, and the highest conductivity at 30 °C was achieved when 40 wt.% of ionic liquid was added. Furthermore, adding Al_2_O_3_ nanoparticles enhanced the mechanical stability of the GPEs, making them more robust. These GPEs with Al_2_O_3_ nanoparticles exhibited good mechanical stability, allowing for the incorporation of more ionic liquid electrolyte (up to 50 wt.%) while still retaining useful mechanical properties. This improved stability enabled a further enhancement in ionic conductivity. Interestingly, when PDADMA TFSI was added to the ionic liquids, the diffusion coefficient of both Li^+^ and anions decreased. However, the decrease in anion diffusion was more significant, resulting in a higher lithium-ion transport number (as evaluated by NMR) than that observed in the original ionic liquids. This indicated that introducing PDADMA TFSI enhanced lithium-ion transport, which is crucial for battery applications. Finally, Forsyth et al. successfully fabricated a highly conductive free-standing GPE membrane demonstrating extremely stable lithium symmetrical cell performance, highlighting the potential of this novel GPE for practical lithium-based battery applications.

#### 2.2.1. Physical Synthesis Processes of GPEs

(i) Synthetic strategies:

Physical preparation methods are commonly employed to fabricate gel-based polymer electrolytes (GPEs). These methods involve physically mixing and manipulating the components to form a homogeneous gel structure. Physical synthesis processes for GPEs offer unique advantages and disadvantages in developing advanced energy storage systems. On the positive side, physical methods are often simpler and more straightforward than chemical ones. They typically involve physically mixing a polymer matrix with a liquid electrolyte and additives, making them accessible and cost-effective. This simplicity in synthesis can also lead to better scalability and reproducibility, which is essential for mass production in the battery industry.

Furthermore, physical synthesis allows for incorporating a wide range of electrolyte materials, enhancing the versatility and adaptability of GPEs for various applications. However, these advantages come with certain limitations. Physical synthesis methods may struggle to achieve precise control over the distribution and interaction of components within the GPE. This lack of control can lead to phase separation, uneven ion transport, and reduced overall performance. Additionally, physical synthesis processes may not be suitable for creating complex or tailored structures essential for achieving high ionic conductivity and stability. Achieving the desired balance between mechanical strength, flexibility, and ionic conductivity can be challenging using purely physical methods. As a result, while physical synthesis offers simplicity and cost-efficiency, it may require additional steps or additives to overcome these limitations and achieve the desired performance characteristics in GPEs. Researchers continue to explore hybrid approaches that combine physical and chemical synthesis techniques to address these challenges and unlock the full potential of GPEs in advanced battery technologies. Some of the key physical preparation methods for GPEs are described below.

Solution Casting: In this method, the polymer and liquid electrolyte components are dissolved in a common solvent to create a homogeneous solution. The solution is then cast onto a substrate and allowed to evaporate, forming a solid gel electrolyte film. The solvent selection is crucial to ensure compatibility with the polymer and electrolyte components [82].

Hot Pressing: Hot pressing involves placing the polymer and liquid electrolyte mixture between two heated plates and applying pressure. The combination of heat and pressure promotes the diffusion and intermolecular entanglement of the polymer chains, leading to gel formation. This method is particularly effective for polymers with low T_g_, which can be molded into a gel state [83].

Freeze–Thaw Method: This method utilizes repeated freezing and thawing cycles to induce gelation. Initially, the polymer and liquid electrolyte are mixed and cooled to a low temperature, causing the formation of small ice crystals. Subsequent thawing breaks the ice crystals, resulting in the formation of a gel structure. This process is repeated several times to enhance gel formation and homogeneity [84].

Solvent Evaporation: This method involves dissolving the polymer in a volatile solvent and mixing it with the liquid electrolyte. The mixture is then allowed to stand, enabling the solvent to evaporate gradually. As the solvent evaporates, the polymer chains start to entangle, forming a gel structure. The choice of solvent is critical to ensure compatibility with the polymer and electrolyte components.

(ii) Properties:

These physical preparation methods offer advantages such as simplicity, scalability, and control over the gel morphology and composition. However, it is essential to consider factors such as selecting appropriate polymers, liquid electrolytes, and solvents to ensure compatibility and optimize the properties of the resulting GPEs. Overall, physical preparation methods provide versatile techniques for fabricating gel-based polymer electrolytes, allowing for the customization of gel structures and properties to meet the requirements of various energy storage applications. Physical preparation methods of GPEs offer several advantages that favor their applications in LIBs. These methods contribute to developing GPEs with desirable properties, leading to improved battery performance and safety. Here are some ways in which physical preparation methods benefit GPE applications in LIBs:

Homogeneous Gel Formation: Physical preparation methods enable the creation of GPEs with a homogeneous gel structure. This uniform distribution of the polymer and electrolyte components ensures efficient ion transport throughout the electrolyte, enhancing the battery’s ionic conductivity. The homogeneous gel structure also promotes good contact between the electrolyte and electrode materials, improving electrochemical performance.

Tailored Composition: Physical preparation methods allow for the precise control of the composition of GPEs. By adjusting the ratios and concentrations of the polymer and electrolyte components, the properties of the GPEs can be tailored to meet specific battery requirements. This customization includes optimizing the ionic conductivity, mechanical strength, and electrochemical stability, enhancing battery performance.

Mechanical Stability: GPEs prepared using physical methods exhibit excellent mechanical stability. The components’ physical mixing and manipulation promote strong intermolecular interactions within the gel structure, such as entanglement or cross-linking. This mechanical stability helps mitigate the mechanical stress caused by volume changes during charge-discharge cycles, preventing electrode damage and prolonging the battery’s lifespan.

Improved Safety: Physical preparation methods facilitate the immobilization of the liquid electrolyte within the gel matrix. This immobilization reduces the risk of electrolyte leakage and the formation of lithium dendrites, which can lead to short circuits and thermal runaway. By enhancing safety, GPEs contribute to LIBs’ overall stability and reliability.

Scalability and Processability: Physical preparation methods are often scalable and compatible with large-scale manufacturing processes. Techniques such as solution casting, hot pressing, and solvent evaporation can be easily adapted to produce GPEs in bulk. This scalability and processability make GPEs prepared via physical methods suitable for commercial battery production.

#### 2.2.2. Chemical Synthesis Processes of GPEs

(i) Synthetic strategies:

In situ chemical preparation methods are widely employed for fabricating GPEs used in LIBs. These methods involve synthesizing and cross-linking polymer networks within the electrolyte system, forming gel structures. In situ chemical preparation methods offer several advantages and are highly relevant to developing GPEs for LIBs. Chemical synthesis processes for GPEs present several advantages and disadvantages in the quest for improved energy storage solutions. On the positive side, chemical methods offer precise control over the electrolyte’s composition, structure, and properties, allowing for the tailored design of materials with specific performance characteristics. This level of control enables the optimization of ionic conductivity, mechanical strength, and thermal stability, which are critical for enhancing the overall performance and safety of batteries. Chemical synthesis also allows for incorporating various functional additives and nanoparticles, further fine-tuning the properties of the GPE to meet the demands of specific applications. However, these advantages come with some notable drawbacks. Chemical synthesis processes tend to be more complex and require careful handling of reactive chemicals, which can lead to increased production costs and safety concerns.

Additionally, achieving reproducibility and scalability in chemical synthesis can be challenging, particularly for advanced materials with intricate structures. Moreover, some chemical reactions used in synthesis may produce byproducts or impurities that need to be carefully managed to avoid detrimental effects on battery performance. Furthermore, optimizing GPEs through chemical methods often involves a trade-off between different properties, making it challenging to achieve a perfect balance between ionic conductivity, mechanical strength, and other critical factors. It has been concluded that chemical synthesis processes offer significant advantages in terms of precise control and customization of GPEs, enabling the development of high-performance energy storage systems. However, their complexity, cost, safety considerations, and the need for careful management of byproducts make them a more intricate choice compared to physical synthesis methods. Here are some key techniques used in in situ chemical preparation:

Polymerization: Polymerization is a standard method used to prepare GPEs. It involves the synthesis of polymer networks within the liquid electrolyte system. Monomers and cross-linkers are mixed with the liquid electrolyte, and a polymerization initiator is added to initiate the cross-linking reaction. Polymerization can be achieved through various techniques such as thermal initiation, photopolymerization, or redox-initiated reactions. The polymerization process forms a three-dimensional network structure, leading to the formation of a gel electrolyte [85].

Cross-Linking Reactions: Cross-linking is another important in situ chemical method used for GPE synthesis. Cross-linking agents or multifunctional monomers are introduced into the liquid electrolyte, which undergoes chemical reactions to form covalent bonds and cross-link the polymer chains. Common cross-linking reactions include condensation, Michael addition, and radical polymerization reactions. The cross-linking process strengthens the polymer matrix, improving the mechanical stability and overall performance of the GPE [86].

In situ Polymerization: In situ polymerization involves the synthesis of the polymer matrix directly within the battery system. This method is typically achieved by introducing monomers, cross-linkers, and polymerization initiators into the battery cell or electrode assembly. The polymerization reaction occurs during the initial charging cycles, forming a gel electrolyte in situ. In situ polymerization ensures intimate contact between the electrolyte and electrode materials, facilitating efficient charge transfer and enhancing battery performance [87].

Interpenetrating Polymer Networks (IPNs): IPNs are formed by simultaneously cross-linking two or more polymers within the liquid electrolyte. This method combines different polymers with complementary properties to create a synergistic effect, enhancing mechanical strength, ionic conductivity, and electrochemical stability. The simultaneous cross-linking of multiple polymers in the liquid electrolyte leads to the formation of a highly interconnected network structure, improving the overall performance of the GPE. In situ chemical preparation methods offer several advantages for GPEs in LIBs. They allow for customizing the gel electrolyte properties by selecting appropriate monomers, cross-linkers, and polymerization conditions. The resulting GPEs exhibit improved ionic conductivity, mechanical stability, electrochemical stability, and compatibility with electrode materials. Additionally, in situ chemical methods enable the fabrication of GPEs directly within the battery system, ensuring good contact and compatibility with the electrodes [88,89,90].

In these typical methods, the precursor solution is formed by dissolving initiators, cross-linkers, and monomers in a liquid electrolyte at a specific ratio. The monomers undergo polymerization under specific conditions, initiating the formation of cross-linking polymer networks. The resulting structure immobilizes the liquid electrolyte uniformly within the nanopores, yielding the GPEs. In situ synthesis commonly employs acrylic esters and ethylene oxide as monomers or cross-linkers. Azo-type (e.g., azo di iso butyro nitrile) or peroxide-type (e.g., benzoyl peroxide) compounds are frequently used as initiators. The chemical bonds formed in the strong cross-linking structure of GPEs prepared through in situ synthesis methods impart excellent thermal stability, preventing solvent leakage even at high temperatures or over prolonged aging periods. Additionally, in situ synthesis methods offer a simple and cost-effective approach to fabricating Li-based batteries with GPEs, as the GPE preparation and solid-state Li-based battery fabrication can be accomplished in a single step. In situ polymerization processes typically involve thermal, radiation, electrochemical, or similar initiation methods.

The in situ thermal-initiated synthesis method is a valuable technique for fabricating GPEs used in various applications, including LIBs. This method involves initiating the polymerization reaction through the application of heat [91]. To begin the process, a precursor solution is prepared by dissolving monomers, cross-linkers, and initiators in a liquid electrolyte. The ratios of these components are carefully controlled to achieve the desired properties of the GPE. The precursor solution is then subjected to thermal treatment, typically through heating. The application of heat serves as the initiator for the polymerization process. As the temperature rises, the monomers undergo chemical bonding and cross-linking, forming a three-dimensional polymer network. This network structure immobilizes the liquid electrolyte within its nanopores, creating the gel-like consistency of the GPE. One of the key advantages of the in situ thermal-initiated synthesis method is its ability to achieve excellent thermal stability. The strong cross-linking structure formed through chemical bonds imparts stability to the GPE, making it resistant to solvent leakage, even at high temperatures or during extended periods of aging.

**Figure 6:**
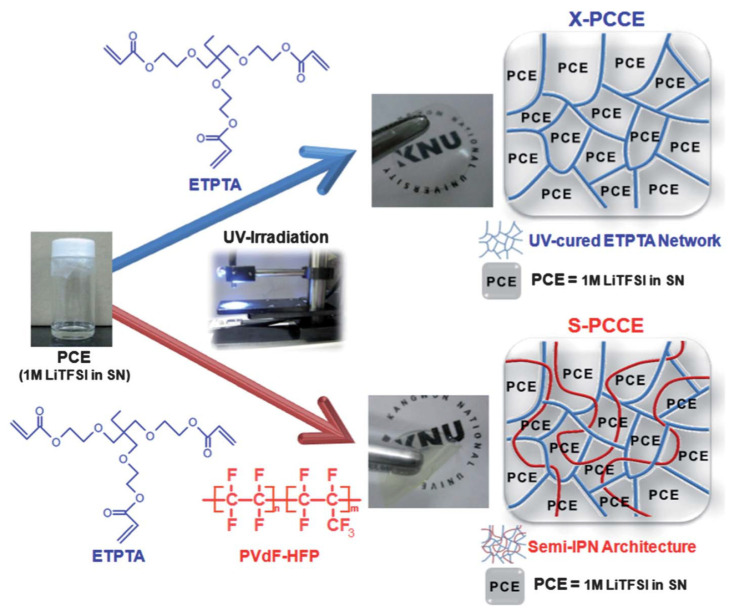
Photos showing the physical characteristics of PCE, X-PCCE, and SPCCE and schematic illustration of the UV-curing process [80]. Reproduced with permission from the Royal Society of Chemistry.

The in situ radiation-initiated synthesis method is a technique used for the fabrication of GPEs. This method involves initiating the polymerization reaction through exposure to radiation, such as ultraviolet (UV) light or gamma radiation. The precursor solution, containing monomers, cross-linkers, initiators, and a liquid electrolyte, is prepared and then exposed to the radiation source. The radiation energy triggers the polymerization process, forming a cross-linked polymer network that immobilizes the electrolyte. The in situ radiation-initiated synthesis method allows for precise control over gel structure and properties, offering potential advantages for developing GPEs for various applications. Ha et al. developed a flexible GPE by combining a semi-interpenetrating polymer network (semi-IPN) matrix and a 1 M LiTFSI in SN plastic crystal electrolyte [79]. The semi-IPN matrix consisted of a UV-cross-linked ethoxylated tri methylol propane tri acrylate (ETPTA) polymer network and a linear polymer PVDF-HFP. Figure 6 represents the schematic diagram of UV curing process. This combination synergistically enhanced the mechanical strength and flexibility of the GPE. The GPE exhibited facile ionic transport, with an impressive ionic conductivity exceeding 10^−3^ S cm^−1^ at room temperature. The steady electrolyte/electrode contact also helped this GPE to operate well in LIBs. However, the in situ radiation-initiated synthesis process simplifies GPE fabrication and yields batteries with favorable electrochemical performance. Its widespread application is limited due to high safety requirements during production.

The in situ electrochemical-initiated synthesis method is a technique used for the fabrication of GPEs in LIBs. This method involves initiating the polymerization reaction through electrochemical means, typically by applying an electric potential or current. In the electrochemical-initiated synthesis process, a precursor solution containing monomers, cross-linkers, initiators, and a liquid electrolyte is prepared. The precursor solution is then electrochemically treated by applying a voltage or current through the electrodes immersed in the solution. The applied electrical potential or current triggers redox reactions, leading to the generation of reactive species that initiate the polymerization of monomers. As the polymerization progresses, a cross-linked polymer network forms, immobilizing the liquid electrolyte and forming the gel-like structure of the GPE. This method offers several advantages. It enables the GPE construction process to be integrated directly into the battery system, ensuring optimal contact and material compatibility between the electrolyte and electrodes. This method also allows the precise control of gel properties by adjusting the electrochemical conditions.

(ii) Properties:

Furthermore, the electrochemical-initiated synthesis method offers the potential for the in situ formation of solid-state interfaces, enhancing the stability and performance of the GPE in the battery system. It also allows for the synthesis of GPEs with tailored properties, such as high ionic conductivity and improved electrochemical stability. Wei et al. prepared a stable GPE layer through the in situ electrochemical-initiated synthesis method [92]. They dissolved 20 wt.% of 1,3-di allyl imidazolium perchlorate (DAIM) monomer into a 1 M EC/PC-Na-ClO_4_ electrolyte. Subsequently, they polymerized the DAIM molecules on the surface of sodium metal electrodes at a specific current density. This process resulted in the formation of a stable GPE layer with a thickness of approximately 50 nm directly on the electrode surface. The in situ formation of this GPE layer proved to be highly advantageous, as it effectively suppressed dendrite growth during sodium deposition, significantly enhancing sodium-metal batteries’ cycling performance. However, it is worth noting that exploring suitable monomers remains a critical aspect in further developing the in situ electrochemical-initiated synthesis method. Identifying and utilizing appropriate monomers with desired properties will continue to be a key focus to optimize the performance and stability of GPEs in electrochemical energy storage systems.

## 3. Potential Applications of Polymer Electrolytes in Li-Based Batteries

Polymer electrolytes have garnered substantial attention in the realm of lithium-based batteries, offering a unique set of advantages and disadvantages that can significantly impact their potential applications. On the positive side, polymer electrolytes have the potential to revolutionize battery safety. Unlike liquid electrolytes, which are highly flammable and can pose serious safety risks, polymer electrolytes are non-flammable, reducing the likelihood of battery-related fires and explosions. This enhanced safety is especially critical in applications where batteries are subjected to physical stress or high-impact situations, such as in electric vehicles or portable electronics. Furthermore, the flexibility and versatility of polymer electrolytes open up a world of possibilities for novel battery designs. These materials can be tailored to possess various mechanical properties, including flexibility and stretchability, making them suitable for use in flexible and wearable electronics. This flexibility allows for the creation of batteries that conform to irregular shapes and can be integrated seamlessly into clothing and other non-traditional form factors. Advancements in polymer chemistry have also led to polymer electrolytes with improved ionic conductivity. Although they still lag behind liquid electrolytes in this regard, these materials have made significant strides, enabling their use in a wide range of portable electronic devices.

Additionally, some polymer electrolytes can operate at higher voltages than traditional liquid electrolytes, potentially increasing the energy density of lithium-based batteries. Another critical advantage of polymer electrolytes is their ability to reduce the growth of lithium dendrites. Dendrites are undesirable needle-like structures that can form on the surface of the lithium anode, leading to short circuits and potentially catastrophic failures. Polymer electrolytes can help inhibit dendrite growth, improving the cycle life and overall reliability of lithium-based batteries. From an environmental standpoint, many polymer electrolytes can be produced using sustainable materials and processes, aligning with the growing demand for eco-friendly battery technologies. This aspect is particularly important as society becomes increasingly conscious of the environmental impact of energy storage solutions. However, it is essential to acknowledge the challenges and disadvantages associated with polymer electrolytes.

One of the primary drawbacks is their lower ionic conductivity compared to liquid electrolytes. This limitation can result in reduced power output and may necessitate larger battery sizes to achieve comparable performance, which can be problematic in applications with stringent space constraints. Additionally, polymer electrolytes often exhibit a narrower operating temperature range than liquid electrolytes. Extreme temperatures can adversely affect their performance, limiting their suitability for applications in harsh environments or regions with extreme climates. The complex manufacturing processes required to produce high-performance polymer electrolytes can be a significant barrier to widespread adoption. Precise control over the composition and structure of these materials is necessary, increasing production costs and complexity. Some polymer electrolytes may also exhibit thermal instability at high temperatures, which can degrade their performance and reliability. Moreover, not all electrode materials are compatible with polymer electrolytes, limiting their applicability in certain types of lithium-based batteries. Lastly, the materials used in polymer electrolytes can be more expensive than traditional liquid electrolytes, contributing to higher overall battery production costs. Overall, polymer electrolytes offer substantial advantages in terms of safety, flexibility, and potential environmental benefits, making them an attractive option for lithium-based battery applications. However, their lower ionic conductivity, limited temperature range, complex manufacturing processes, potential thermal instability, material compatibility issues, and higher production costs represent significant challenges that researchers are actively working to address. As these challenges are overcome, polymer electrolytes have the potential to play a pivotal role in shaping the future of energy storage technology.

In LIBs, polymer electrolytes enable the development of solid-state cells, eliminating the need for flammable liquid electrolytes and enabling the use of higher energy density electrode materials. Additionally, polymer electrolytes find potential applications in Li-air, Li-sulfur, and solid-state Li batteries. Their ability to accommodate different lithium salts, plasticizers, and fillers opens doors for customization and optimization, driving innovation in advanced energy storage systems. These different types of LIBs with polymer electrolytes demonstrate the versatility and potential of polymer electrolytes in enabling advancements in energy storage technologies across various industries.

Lithium-Ion Polymer Batteries (LiPo): These batteries utilize polymer electrolytes as solid or gel-like materials that offer flexibility in battery design. Due to their thin and lightweight form factor, LiPo batteries find applications in portable electronic devices such as smartphones, tablets, and wearable devices. In a study by Lee et al. [93], the effect of unreacted monomer on the functionality of LiPo batteries was investigated. The polymer electrolytes used in the batteries are made of cross-linked polyethylene glycol diacrylate and are created by free radical polymerization. As the proportion of unreacted monomer in the electrolytes increases, particularly at low temperatures and high discharge rates, the discharge capacities of test cells containing polymer electrolytes made with various initiator concentrations drop. This behavior indicates a correlation between the increase in unreacted monomer content and the rise in interfacial resistance within the test cells. The cause of this high interfacial resistance is believed to be the reaction of the unreacted monomer at the electrode surface during the charging process, resulting in the formation of a resistive film. In another work, polymer electrolytes for Li-ion polymer batteries were investigated using various combinations of lithium difluoro(oxalato)borate (LiODFB) and lithium bis(oxalate)borate (LiBOB) salts, along with an ionic liquid (N-methyl-N-propyl pyrrolidinium bis(trifluoro methane sulphonyl)imide or 1-ethyl-3-methyl imidazolium bis(tri fluoro methane sulphonyl)imide, sulfolane (TMS), and poly(vinylidene fluoride). The preparation of polymer electrolytes involved combining quaternary lithium salt, ionic liquid, PVdF, and TMS using the casting technique [94].

Lithium-Ion Solid-State Batteries: Solid-state batteries employ polymer electrolytes as a solid material, eliminating the need for flammable liquid electrolytes. They offer improved safety, higher energy density, and longer cycle life. Solid-state batteries are considered for electric vehicles (EVs) and grid energy storage due to their enhanced stability and potential for increased energy storage capacity. A novel design for SPEs was proposed by Wang et al., utilizing electrospinning to create an ultrathin and rigid blend [95]. The blend consisted of a bio-polyamide with an N-substituted pyrrolidone ring (referred to as IBD) and PEO/Li bis(tri fluoro methane sulfonyl)imide. The experimental results confirmed that IBD exhibited a strong affinity for Li^+^ ions, making it the primary component responsible for ion transport in the SPE. The combination of IBD with the flexible chain segments of PEO resulted in a synergistic effect, facilitating the transport of ions. This mechanism involved IBD promoting the dissociation of ion pairs, leading to dynamic interactions between the mobile cations and the long-chain molecules that constituted the SPE.

Additionally, this combination contributed to the widening of the ion transport pathway. The resulting SPE exhibited an impressive ionic conductivity of up to 4.26 × 10^−4^ S cm^−1^ at 50 °C. In another work, Zhu et al. proposed a novel design of inorganic-polymer gel electrolyte/anode interphase in quasi-solid-state LIBs [96]. This study aimed to develop a gel electrolyte and anode electrode employing helical mesoporous silica nanofibers (HMSFs) as the underlying structure to improve the electrochemical performance of LIBs. This innovative approach allows for the seamless integration of the electrolyte and anode interface, resulting in remarkable electrochemical properties, as observed through measurements. Several noteworthy characteristics were achieved by incorporating HMSFs into a P(VDF-HFP) matrix to form electrolyte membranes referred to as NPCGE. These included exceptional thermal stability, withstanding temperatures of up to 372 °C, a wide electrochemical window of 5.30 V, and a high room temperature ionic conductivity of 1.2 × 10^−3^ S cm^−1^.

**Figure 7:**
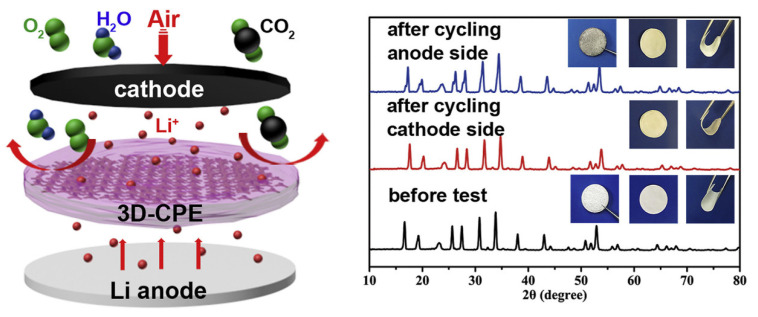
Schematic diagram of 3D garnet network composite polymer electrolyte [98]. Reproduced with permission from Elsevier.

Lithium-Air Batteries: Li-air batteries, also known as lithium-oxygen batteries, are designed to utilize oxygen from the surrounding air as a cathode material. Polymer electrolytes play a crucial role in these batteries by providing a stable and conductive medium for lithium-ion transport. Lithium-air batteries have the potential to offer high energy density and could be used in electric vehicles and aerospace applications. Yoon et al. recently proposed a new GPE for Li-air batteries with excellent ionic conductivity, lithium stability, and redox activity [97]. The introduction of IL-PTZ in Li-air cells leads to significantly improved electrochemical performance. The cell exhibits a four-times-longer cyclability than the cell without the addition and a remarkable initial discharge capacity of 4685 mAh g^−1^. Previously, Song et al. revealed that the performance of solid-state lithium-air batteries is greatly improved by including a continuous 3D garnet network composite polymer electrolyte (Figure 7) [98,99].

Lithium-Sulfur Batteries: Polymer electrolytes can also be used in lithium-sulfur batteries, which utilize a sulfur-based cathode. These batteries offer high energy density and are being explored for applications in electric vehicles, renewable energy storage, and portable electronics. Hui et al. reported on a cathode material and additive called poly(sulfur-1,3-diisopropenylbenzene) (PSD), based on an organosulfide, was synthesized, and incorporated into a P(VDF-HFP) polymer electrolyte. The resulting composite, named P(VDF-HFP)-10%PSD, exhibited superior ionic conductivities compared to the liquid electrolyte, reaching up to 2.27 × 10^−3^ S cm^−1^ at room temperature [100]. In another work, a novel flame-retardant polymer electrolyte was developed to improve the Li-ion conductivity, aiming to enhance the safety of lithium-sulfur batteries [101]. The same group of researchers introduced a pioneering approach of in situ electrochemical polymerization to fabricate a nonflammable polyether electrolyte in Li-S@pPAN batteries [102]. This innovative technique enhances lithium compatibility (exceeding 3000 h) and modifies the cathode interphase, improving reaction kinetics. The resulting polyether-rich interphase ensures remarkable battery performance, including a higher capacity of 1645.3 mAh g^−1^ based on S, faster charging capability (up to 10 °C), and exceptional cycling stability (maintaining 99.5% retention over 400 cycles).

**Figure 8:**
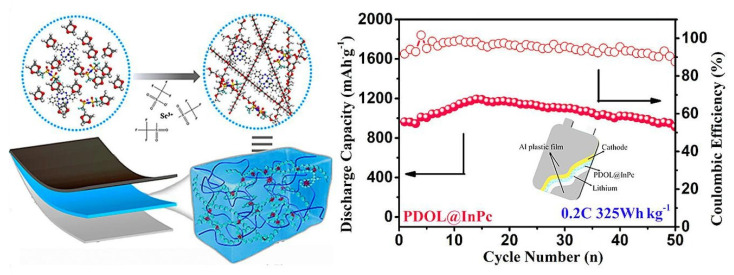
Schematic development of a gel electrolyte modified with InPc through in situ polymerization for high-loading Li–sulfur batteries and their electrochemical performance [102]. Reproduced with permission from Elsevier.

Moreover, it achieved an ultrahigh average Coulombic efficiency (CE) of over 99.9995%, indicating minimal irreversible reactions on the cathode. In a pouch cell configuration, the battery exhibited a high sulfur utilization rate of 91.2% and maintained stable performance with 84.6% capacity retention over 60 cycles. A novel SPE was developed in recent work through the insitu polymerization of 1,3-dioxolane (DOL) in the presence of a multifunctional indium phthalocyanine (PDOL@InPc) (Figure 8) [102]. Including InPc in the SPE enhanced ionic conductivity and effectively mitigated the shuttle effect. Furthermore, InPc served as an effective additive that facilitated uniform lithium deposition by restructuring the electric field at the interface.

Lithium-Metal Batteries: Li-metal batteries are an advanced battery technology that utilizes lithium metal as the anode material. These batteries have the potential to offer extremely high energy density, making them attractive for applications that require long-lasting and high-performance energy storage solutions. Still, the development of Li metal batteries faces safety challenges, as using pure lithium metal can lead to dendrite formation and potential short circuits. Efforts are underway to incorporate polymer electrolytes and solid-state designs to enhance the safety and stability of lithium metal batteries, opening up possibilities for next-generation energy storage systems with even greater capacity. Xie et al. recently introduced a new kind of GPE, named AT11-GF, by using adiponitrile (ADN), a nonflammable substance that conducts Li^+^ ions effectively. This electrolyte shows great potential for solid-state lithium (Li)-metal batteries [103]. To address the issue of ADN and triethyl phosphate (TEP) causing unwanted reactions on the surface of the Li anode, fluoroethylene carbonate (FEC) was added as a film-forming agent. This addition helps create a LiF-enriched interface within the ADN-based electrolyte system. The resulting electrolyte demonstrated a high ionic conductivity of 1.724 × 10^−3^ S cm^−1^ at room temperature, a wide electrochemical window of approximately 5.75 V compared to Li^+^/Li, a favorable Li^+^ ion transfer number (t_Li+_ =0.70), and excellent compatibility with the Li anode (remaining stable even at a current density of 0.2 mA cm^−2^ for 1000 h).

A new family of block copolymer polyelectrolytes with single-ion conducting properties was successfully synthesized using the reversible addition–fragmentation chain transfer polymerization technique. These copolymers consist of blocks of poly(lithium 1-[3-(methacryloyloxy)propylsulfonyl]-1-(trifluoromethylsulfonyl)imide) and poly(ethylene glycol) methyl ether methacrylate. The resulting polyelectrolytes exhibit a range of low T_g_ values from −61 to 0.6 °C, demonstrating high ionic conductivity at different temperatures (up to 2.3 × 10^–6^ and 1.2 × 10^–5^ S cm^–1^ at 25 and 55 °C, respectively). Additionally, they demonstrate wide electrochemical stability, withstanding potentials up to 4.5 V vs. Li^+^/Li. Furthermore, they possess a lithium-ion transference number close to unity, specifically 0.83. Due to the combination of these exceptional properties, the prepared polymer materials have been employed as solid polyelectrolytes and binders to develop lithium-metal battery prototypes. These prototypes display remarkable charge/discharge efficiency and outstanding specific capacity (up to 130 mAh g^–1^) at a C/15 rate [104].

In another work, Porcarelli et al. successfully developed an innovative polymer electrolyte system by controlling the mobility of traditional EO-based backbones. This newly designed polymer electrolyte enables the creation of all-solid lithium-based polymer cells, exhibiting remarkable cycling performance in both rate capability and stability across a wide range of operating temperatures. The polymer electrolytes were synthesized through UV-induced (co)polymerization, facilitating effective interlinking between the PEO chains, which are plasticized by tetraglyme at various lithium salt concentrations. The resulting polymer networks demonstrate exceptional mechanical robustness, high flexibility, and exhibit a homogeneous and highly amorphous structure. These polymer electrolytes exhibit impressive ionic conductivity values exceeding 0.1 mS cm^−1^ at ambient temperatures. They also display a wide electrochemical stability window, capable of withstanding potentials greater than 5 V vs. Li^+^/Li. Furthermore, these electrolytes possess an excellent lithium-ion transference number, exceeding 0.6, and display favorable interfacial stability. An important advantage of these polymer electrolytes is their effective resistance to the nucleation and growth of lithium dendrites. This property makes them highly promising for utilization in the next generation of all-solid Li-metal batteries operating under ambient conditions [105]. Overall, Porcarelli et al. demonstrated the successful architecture of a novel polymer electrolyte system with impressive properties, paving the way for enhanced performance and safety in advanced all-solid lithium-based batteries.

## 4. Conclusions

Polymer electrolytes hold immense promise for revolutionizing the field of lithium-ion (Li-ion) batteries, offering numerous advantages such as improved safety, higher energy density, and enhanced stability. As research and development efforts continue, the prospects of polymer electrolytes for LIBs look promising. However, several challenges still need to be addressed for their widespread application. One of the key future prospects of polymer electrolytes lies in their potential to enable the development of all-solid-state batteries. By replacing flammable liquid electrolytes with SPEs, these batteries can significantly enhance safety and energy density. All-solid-state batteries also hold promise for applications in electric vehicles, portable electronics, and grid-scale energy storage due to their improved stability and high energy storage capacity.

Furthermore, using polymer electrolytes allows for the utilization of lithium metal anodes, which offer higher theoretical capacities than traditional graphite anodes. This enables the development of Li-metal batteries with improved energy density and performance. Additionally, polymer electrolytes can be tailored to exhibit high ionic conductivity, crucial for enhancing LIBs’ power output and overall efficiency. Despite the promising prospects, challenges persist in the application of polymer electrolytes. One of the significant challenges is achieving high ionic conductivity while maintaining mechanical stability. Improving the ion transport properties of polymer electrolytes without compromising their mechanical strength is crucial for enabling efficient battery performance and long cycle life. Researchers are exploring various strategies, such as nanocomposite structures and polymer blending techniques, to enhance the ionic conductivity and mechanical properties of polymer electrolytes.

Moreover, ensuring the long-term stability and compatibility of polymer electrolytes with other battery components remains challenging. Compatibility issues can arise between the polymer electrolyte and electrodes or separators, leading to degradation and reduced battery performance. Addressing these challenges requires a comprehensive understanding of the chemical and electrochemical interactions within the battery system, as well as the development of advanced materials and manufacturing processes. In conclusion, the prospects of polymer electrolytes in various types of LIBs are highly promising. They offer improved safety, higher energy density, and enhanced stability. However, ionic conductivity, mechanical stability, and compatibility challenges must be overcome through continued research and development efforts. With advancements in materials science, manufacturing techniques, and battery design, polymer electrolytes have the potential to play a vital role in the next generation of high-performance LIBs, contributing to the advancement of renewable energy storage, electric mobility, and other emerging applications.

## Figures and Tables

**Table 1:** Studies on solid polymer electrolytes (SPEs) and their significant properties [24]. Reproduced with permission from Springer Nature.

Polymer	Salt	Filler/Plasticizer	Solvent	Conductivity (S cm^−1^)	Thermal Stability (°C)	ESW (V)	Source
PVA	Mg(NO_3_)_2_	Triple distilled water		7.36 × 10^–7^			[34]
PAN	LiClO_4_	ALA-MMT	DMF	2.44 × 10^−4^	4.75	-	[35]
PVC-PEMA	LiPF_6_	THF	-	10^−3^	4.3	-	[36]
PEO	LiTf	EC/sepiolite	-	10^−4^	4.5	-	[37]
PEO-PMMA	LiClO_4_	PA	NMP	1.59 × 10^−5^	209	>5	[38]
PVA-PVdF	LiClO_4_	DMF	-	3.03 × 10^−5^	273	-	[39]
PEO-PMMA	LiTFSI	Al_2_O_3_	THF	3.24 × 10^−4^	4.6	-	[40]
PVdF-PVA	LiTFSI	MMT	DMF	4.31 × 10^−4^	4.7	-	[41]
PAN	LiPF_6_	DMMT	DMF	3.7 × 10^−3^	200	4.3	[42]
PAN	LiCF_3_SO_3_	DMMT	DMF	10^−3^	250	5.6	[43]
PEO	LiClO_4_	SiO_2_	-	1.2 × 10^−3^	-	5.5	[44]
PEO	LiAsF_6_	MMT	ACN	4 × 10^−5^	-	-	[45]
PEO	LiCF_3_SO_3_	Al_2_O_3_-DOP	-	7.60 × 10^−4^	-	-	[46]
PAN-PVA	LiClO_4_	EC/DMC	EC/DMC	2.5 × 10^−4^	300	4.8	[47]
PVA-PVdF	LiCF_3_SO_3_	DMF	-	2.7 × 10^−3^	175	3.4	[48]
PEO	LiFSI	ACN	-	1.3 × 10^−3^	-	5.7	[49]
PVA	NH_4_Al(SO_4_)_2_12H_2_O	Water		1.73 × 10^−4^	∼350	-	[50]
PVC-PEMA	LiClO_4_	PC	THF	3.45 × 10^−3^	230	-	[51]
PVA	Cu(NO_3_)_2_	-	-	1.6 × 10^−5^	-	-	[52]
PMMA	LiClO_4_	PC	-	7.04 × 10^−3^	∼240	-	[53]
PVB	LiClO_4_	PEG	-	2.15 × 10^−6^	-	-	[54]
PVdF-PAN	NaHCO_3_	SiO_2_	-	3.32 × 10^−3^	-	5	[55]

## Data Availability

The data presented in this study are available on request from the corresponding author.
